# Prediction for Breast Cancer in BI-RADS Category 4 Lesion Categorized by Age and Breast Composition of Women in Songklanagarind Hospital

**DOI:** 10.31557/APJCP.2021.22.2.531

**Published:** 2021-02

**Authors:** Seechad Noonpradej, Piyanun Wangkulangkul, Piyanoot Woodtichartpreecha, Suphawat Laohawiriyakamol

**Affiliations:** 1 *Division of General Surgery, Faculty of Medicine, Songklanagarind hospital. Prince of Songkla University, Songkla, Thailand. *; 2 *Division of Radiology, Faculty of Medicine, Songklanagarind Hospital, Prince of Songkla University, Songkhla, Thailand. *

**Keywords:** Age, BI-RADS category 4, breast cancer, breast composition, prediction

## Abstract

**Background:**

Older age and dense breast are the important risk factors for breast cancer. The ACR BI-RADS lexicon 5^th^ edition does not mention how patient age and breast density may affect the category assessment. The aim of this study was to investigate whether patient age and breast density influence the positive predictive value (PPV) of mammographic and ultrasonographic findings categorized as BI-RADS category 4 and subcategories 4a, 4b, and 4c among female patients.

**Materials and Methods:**

A retrospective study was conducted in Songklanagarind Hospital between January 1, 2016 and December 31, 2017 in female patients older than 18 years who had breast lesions categorized as BI-RADS category 4 and subcategories 4a, 4b, 4c. A total of 961 breast lesions consisted of 772 (80.33%) benign lesions and 189 (19.67%) malignant lesions. Categorization was done in each lesion based on age ranges of ≤35 years, >35 to 60 years, and >60 years and breast density according to mammographic breast composition. The PPV for each BI-RADS category was calculated based on the pathological diagnoses and were compared using the chi-square test.

**Results:**

The overall PPV in each subcategory was in the reference range. The PPV increased with increasing age: 4% vs. 22.63% vs. 36.67% for category 4 (p-value=0.01); 0% vs. 5.81% vs. 6.88% for subcategory 4a (p-value=0.002); 6.67% vs. 26.62% vs. 51.35% for subcategory 4b (p-value=0.001); and 33.33% vs. 76.92% vs. 81.82% for subcategory 4c (p-value=0.02). An association was not found between PPV and breast density.

**Conclusion:**

A significantly positive association was found between PPV and age in patients in BI-RADS subcategories 4a, 4b, and 4c. This study could not determine that mammographic breast composition according to the ACR BI-RADS 5th edition was associated with PPV due to improper sample distribution.

## Introduction

Breast cancer is the most common cancer and the leading cause of cancer-related deaths among women in Songkhla Province and Thailand (Imsamran et al., 2018; Rojanamethin et al., 2020). With greater awareness and easier access to information, a large number of women visit health care centers for breast examinations which has resulted in early detection and a reduction in mortality (Nelson et al., 2016; National Comprehensive Cancer Network, 2020; MD Abu and Arun, 2020). 

The standard mammography report form based from the Breast Imaging Reporting and Data System (BI-RADS) lexicon created by The American College of Radiology (ACR), which was updated to the fifth edition in 2013 (D’Orsi et al., 2013; Spak et al., 2017), is widely accepted because it is easy to use and provides management guidance. The lexicon defines final assessment categories to describe the level of breast cancer suspicion from radiographic findings. Each category has a specific positive predictive value (PPV) and management recommendation that suggest biopsies in subcategories 4a, 4b, and 4c as reported from D’Orsi (2013). A false-positive examination that leads to a negative biopsy causes distress that may be sufficient to deter a woman from attending the next breast cancer screening appointment (Bond et al., 2017). Several different methods and risk prediction models are used to decrease false-positive examinations, but the methods are still controversial (Mazouni et al., 2010; Flowers et al., 2013; McCarthy et al., 2015). 

Older age and dense breast are the important risk factors for breast cancer, especially older than 40 years (Weir et al., 2007; Kotepui and Chupeerach, 2013; Nindrea et al., 2017; Momenimovahed and Salehiniya, 2019), and extremely dense breast tissue (National Breast and Ovarian Cancer Centre, 2009; Pettersson et al., 2014; Bae and Kim, 2016; Momenimovahed and Salehiniya, 2019) which can increase cancer risk by 4 times as reported from National Breast and Ovarian Cancer Centre (2009). However, these two factors are inversely correlated to each other because younger women have denser breasts. The ACR BI-RADS lexicon 5th edition does not mention how patient age and breast density may affect the category assessment. This retrospective study aimed to investigate whether patient age and breast density influence the PPV of the mammographic and ultrasonographic findings categorized as BI-RADS category 4 and subcategories 4a, 4b, and 4c among female patients in southern Thailand who visited Songklanagarind Hospital.

## Materials and Methods

*The study design was approved by:*

The Songklanagarind Hospital Ethics Committee.

*Study population*

Retrospective study which collected data from the Hospital Information System of Songklanagarind Hospital between January 1, 2016 and December 31, 2017. The enrolled subjects were female patients aged ≥18 years who had lesions categorized as category 4, 4a, 4b, or 4c and imaging and official pathological reports for these lesions were available for review. Non-pathological confirmed lesions, which were followed up by imaging until reported as category 2, were classified as benign by imaging. In each lesion, we recorded only the moment when the highest grade was reported.

*Imaging and BI-RADS classification*

In our institution, both mammography (tomosynthesis) and ultrasonography are performed routinely as screening or diagnostic tools, but ultrasonography alone is performed in patients younger than 40 years. The standard protocols for mammography and ultrasonography are used. The findings are reported by one of nine radiologists (two breast radiologists and seven general radiologists) with more than 5 years of experience according to the ACR BI-RADS lexicon 5^th^ edition. Breast density is assessed from mammographic breast composition.

*Pathological report and follow-up*

In our practice, biopsy method depends on the surgeon and the patient. Biopsies are done at the clinic or operating room by a surgeon for palpable lesions. If the lesion cannot be palpated, sonographic-guided or stereotactic-guided core needle biopsy is performed by a radiologist. In this study, the pathological diagnoses of those lesions with initial core needle biopsy and subsequent surgical excision were recorded from the final surgical excision results. In each lesion, the most malignant potential report was recorded as the final pathological diagnosis. Subcategory 4a lesions with clinically low suspicion of malignancy were followed up every 6 months. If the lesion was stable for more than 2 years, it was categorized as BI-RADS 2 and classified as benign by imaging.

*Statistical analysis*

For the purposes of this study, PPV is defined as the number of true positive biopsies from all included examinations. A biopsy was considered true positive if the tissue was diagnosed as cancer. Lesions were classified by age group and breast composition. Age was divided into three groups according to the age of incidence of breast cancer as Kotepui and Chupeerach (2013) reported in previous work, group 1 was the youngest (≤35 years), group 2 was the age of peak incidence (>35 to 60 years), and group 3 was the oldest (>60 years). The categories of breast composition took into account the chance that a mass could be obscured by fibroglandular tissue in mammography and were categorized into four types: (1) entirely fatty; (2) scattered areas of fibroglandular density; (3) heterogeneously dense; and (4) extremely dense. We calculated the PPV in different age groups and breast composition as well as the overall PPV in BI-RADS category 4 and subcategories 4a, 4b, and 4c. The PPV of each BI-RADS category among the age groups and breast composition were compared using the chi-square test. A p-value less than 0.05 was considered statistically significant. Program R version 2.13.1 was used for the statistical analysis.

## Results

Between January 1, 2016 and December 31, 2017, 25,836 breast images were obtained in our institute. A total of 924 patients with 961 breast lesions were included in the study. Benign lesions totaled 772 (80.33%) and malignant lesions totaled 189 (19.67%). Patients with malignant lesions were significantly older than the benign group (57.72±13.24 vs. 44.21±13.27; p-value<0.001). In both groups, most of the patients had heterogeneously dense breast composition (56.1% vs. 65.1%) followed by scattered areas of fibroglandular density (14.1% vs. 24.9%). Breast mass was the most common indication for examination in both groups (34.3% vs. 44.4%) followed by screening (26% vs. 21.7%) (Table 1).

The pathological results of lesions related to the BI-RADS categories are shown in Table 2. Invasive ductal carcinoma was the most common malignant diagnosis (60.31%) and was also most common in each subcategory. Other malignant lesions included ductal carcinoma in situ (23.81%), mucinous carcinoma (4.23%), metastatic tumor (4.23%), invasive lobular carcinoma (3.17%), and other types which included papillary carcinoma, malignant phyllodes tumor, medullary carcinoma, tubular carcinoma, and lymphoma. Invasive ductal carcinoma, invasive lobular carcinoma, and metastatic tumor tended to increase in the higher categories from subcategory 4a to 4c, while ductal carcinoma in situ and mucinous carcinoma did not show this trend.

Among the 772 benign lesions, fibroadenoma (34.46%) was the most frequent histological type (Table 2) which was found mostly in subcategory 4a (43.38%). Fibrocystic change and fibrosis were the benign lesions which were most commonly categorized as BI-RADS 4c (23.8%).

The total PPVs calculated for each BI-RADS category based on the pathological diagnoses were 22.4%, 4.1%, 29.84%, and 76.67% and were in the ACR 2013 reference ranges of category 4 and subcategories 4a, 4b, and 4c, respectively. The age-related PPV of each BI-RADS category varied significantly among all age groups (p-value<0.05) (Table 3). Positive relationships between increasing age and age-related PPV were found: 4% vs. 22.63% vs. 36.67% for category 4 (p-value=0.01); 0% vs. 5.81% vs. 6.88% for subcategory 4a (p-value=0.002); 6.67% vs. 26.62% vs. 51.35% for subcategory 4b (p-value=0.001); and 33.33% vs. 76.92% vs. 81.82% for subcategory 4c (p-value=0.02). Lesions categorized in category 4 resembled the PPV of subcategory 4b in each age group.

The breast composition-related PPV of each BI-RADS category varied significantly among the four breast composition types (p-value<0.05) except subcategory 4c (p-value=0.109) (Table 4). The PPV in each breast composition type was not correlated in the same direction. The likelihood of having cancer may not be correlated with increasing breast density.

**Table 1:** Characteristics of Abnormal Mammography BI-RADS Category 4 Patients in Songklanagarind Hospital

Characteristic	Benign n=772	Malignant n=189	p-value
Mean Age (Year) (SD)	44.21 (13.27)	54.72 (13.24)	0.007
≤35 year (%)	184 (23.8)	3 (1.6)	<0.001
>35 to 60 year (%)	515 (66.7)	136 (72)	
>60 year (%)	73 (9.5)	50 (26.5)	
Median BMI (IQR)	22.8 (20.5,26)	23.6 (21.5,26.7)	0.012
Breast composition (%)			
Entirely fatty	9 (1.2)	9 (4.8)	<0.001
Scattered areas of fibroglandular density	109 (14.1)	47 (24.9)	
Heterogeneously dense	433 (56.1)	123 (65.1)	
Extremely dense	19 (2.5)	7 (3.7)	
No assessment (US only)	202 (26.2)	3 (1.6)	
Indication for examination (%)			
Screening	203 (26.3)	41 (21.7)	<0.001
Follow-up	178 (23.1)	19 (10.1)	
Breast mass	265 (34.3)	84 (44.4)	
Mastalgia	34 (4.4)	6 (3.2)	
Nipple discharge	15 (1.9)	9 (4.8)	
Axillary mass	10 (1.3)	4 (2.1)	
Surveillance	67 (8.7)	24 (12.7)	
Metastasis work up	0 (0)	2 (1.1)	
Mode of examination (%)			
Mammography with US	574 (74.4)	187 (98.9)	<0.001
Ultrasonography	198 (25.6)	2 (1.1)	
BI-RADS categories (%)			
4	149 (19.3)	43 (22.8)	<0.001
4a	468 (60.6)	20 (10.6)	
4b	134 (17.4)	57 (30.2)	
4c	21 (2.7)	69 (36.5)	

Data are shown as n (%) unless indicated otherwise; SD, standard deviation; IQR, interquartile range; BMI, body mass index; US, ultrasonography; BI-RADS, Breast Imaging Reporting and Data System

**Table 2:** Pathological Results of Lesions Divided into BI-RADS Category 4 and Subcategories

Pathological report	B4	B4a	B4b	B4c	Overall
	n=192	n=488	n=191	n=90	n=961
Benign (%)	149 (77.6)	468 (95.9)	134 (70.16)	21 (23.33)	772 (80.33)
Benign by imaging	22 (14.77)	74 (15.81)	9 (6.72)	0 (0%)	105 (13.6)
Fibroadenoma	32 (21.48)	203 (43.38)	30 (22.39)	1 (4.76)	266 (34.46)
Fibrocystic change	18 (12.08)	69 (14.74)	36 (26.86)	5 (23.8)	128 (16.58)
Fibrosis	7 (4.7)	28 (6)	22 (16.42)	5 (23.8)	62 (8.03)
Benign phyllodes tumor	4 (2.68)	2 (0.43)	5 (3.73)	1 (4.76)	12 (1.55)
Epithelial hyperplasia without atypia	5 (3.36)	6 (1.28)	3 (2.24)	0 (0%)	14 (1.81)
Sclerosing adenosis	13 (8.72)	29 (6.2)	14 (10.45)	2 (9.52)	58 (7.51)
Intraductal papilloma	13 (8.72)	20 (4.27)	9 (6.72)	3 (14.29)	45 (5.83)
Acute/chronic inflammation	9 (6.04)	9 (1.9)	2 (1.49)	2 (9.52)	22 (2.85)
Benign fibroepithelial tissue	9 (6.04)	10 (2.1)	1 (0.75)	1 (4.76)	21 (2.72)
Other^a^ benign lesions	17 (11.41)	18 (3.84)	3 (2.24)	1 (4.76)	39 (5.05)
Malignant (%)	43 (22.4)	20 (4.1)	57 (29.84)	69 (76.67)	189 (19.67)
Invasive ductal carcinoma	23 (53.49)	13 (65)	31 (54.39)	47 (68.12)	114 (60.31)
Ductal carcinoma in situ	13 (30.23)	5 (25)	17 (29.82)	10 (14.49)	45 (23.81)
Invasive lobular carcinoma	0 (0)	0 (0)	1 (1.75)	5 (7.25)	6 (3.17)
Mucinous carcinoma	1 (2.33)	2 (10)	3 (5.26)	2 (2.9)	8 (4.23)
Metastatic tumor	4 (9.3)	0 (0)	1 (1.75)	3 (4.35)	8 (4.23)
Other^b^ malignant lesions	1 (2.33)	0 (0)	2 (3.51)	1 (1.45)	8 (4.23)

Data are shown as n (%); ^a^, Includes radial scar, atypical lobular hyperplasia, atypical ductal hyperplasia, lobular carcinoma in situ, abscess, cyst, duct ectasia, reactive lymph node hyperplasia, and fat necrosis; ^b^, Includes papillary carcinoma, malignant phyllodes, medullary carcinoma, tubular carcinoma, and lymphoma

**Table 3:** Age-related Positive Predictive Values of BI-RADS Category 4 and Subcategories

Age	BI-RADS			
	4	4a	4b	4c
Mean (SD)	49.38 (12.44)	41.84 (12.85)	50.87 (11.97)	54.02 (11.11)
Age group (%)				
≤35 year	4 (1/25)	0 (0/144)	6.67 (1/15)	33.33 (1/3)
>35 to 60 year	22.63 (31/137)	5.81 (18/310)	26.62 (31/139)	76.92 (50/65)
>60 year	36.67 (11/30)	6.88 (2/34)	51.35 (19/37)	81.82 (18/22)
Total (%)	22.4 (43/192)	4.1 (20/488)	29.84 (57/191)	76.67 (69/90)
ACR 2013 reference range (%)	>2 to <95	>2 to <10	>10 to ≤50	>50 to <95
p-value	0.01	0.002	0.001	0.02

Data are shown as n (%); BI-RADS, Breast Imaging Reporting and Data System; SD, standard deviation; ACR, American College of Radiology

**Table 4:** Breast Composition-Related Positive Predictive Values of BI-RADS Category 4 and Subcategories

	BI-RADS			
Breast composition (%)	4	4a	4b	4c
Entirely fatty	75 (3/4)	14.29 (1/7)	50 (2/4)	100 (3/3)
Scattered areas of fibroglandular density	22.41 (13/58)	0 (0/44)	48.64 (18/37)	94.11 (16/17)
Heterogeneously dense	24.21 (23/95)	6.15 (16/260)	26.32 (35/133)	72.06 (49/68)
Extremely dense	28.57 (2/7)	20 (3/15)	50 (1/2)	50 (1/2)
No assessment (US only)	7.14 (2/28)	0 (0/162)	6.67 (1/15)	–
Total (%)	22.4 (43/192)	4.1 (20/488)	29.84 (57/191)	76.67 (69/90)
ACR 2013 reference range (%)	>2 to <95	>2 to <10	>10 to ≤50	>50 to <95
p-value	0.032	<0.001	0.007	0.109

Data are shown as n (%); BI-RADS, Breast Imaging Reporting and Data System; US, ultrasonography; ACR, American College of Radiology

## Discussion

According to the ACR BI-RADS lexicon 5th edition, category 4 is divided into three subcategories which have different PPVs: 4a=>2% to <10%, 4b=>10% to ≤50%, and 4c=>50% to <95% (American College of Radiology, 2013). Many previous studies confirmed these values (Wiratkapun et al., 2010; Burivong and Amornvithayacharn, 2011; Lazarus et al., 2011; Yoon et al., 2011; Chaiwerawattana et al., 2012; Elezaby et al., 2018; He et al. 2019) including three retrospective studies from Wiratkapun (2010), Burivong and Amornvithayacharn (2011), and Chaiwerawattana (2012) in Thai women with a sample up to 555 patients. This present study contained the largest population in southern Thailand (n=961) and all values for category 4 and the subcategories (4=22.4%, 4a=4.1%, 4b=29.84%, and 4c=76.67%) were located within the ACR reference ranges.

In our population, the most common malignancy was invasive ductal carcinoma (60.31%) and was also the most common in each category which gradually increased in the higher subcategories (4c > 4b > 4a) followed by ductal carcinoma in situ (23.81%). Fibroadenoma (34.46%) and fibrocystic change (16.58%) were not only the most common benign lesions found in this study but also the most common cause of false-positive results. All of these findings were similar to previous studies as Wiratkapun et al., (2010), FU et al., (2011), Yoon et al., (2011), Hu et al., (2018), and Suttawas (2018). 

In the age-specific analysis of each category, age-related PPV gradually increased in the higher age groups in subcategories 4a–c (all p-values<0.05), which was similar to studies by Hu et al., (2018) and He et al., (2019). It has been reported age-related PPVs in subcategories 4a and 4b in the same way as our study (4a, p-value<0.001 and 4b p-value=0.0139), but a significant difference was not found in subcategory 4c (p-value=0.185) (Fu et al., 2011). In age group 3 (>60 years), the age-related PPV for subcategory 4b (51.35%) exceeded the ACR reference range (>10% to ≤50%). The age-related PPVs in subcategories 4a, 4b, and 4c in age group 1 (≤35 years) were lower than the reference range. This result was possibly due to the low population of these subcategories (15 in 4a and 3 in 4c) which led to a low reliability. Subcategory 4a in age group 1 had a lower than usual age-related PPV (0%) in our study. This result needs careful interpretation because benign lesions from imaging were included and most were in subcategory 4a (70%). As He et al., (2019) reported in previous work, excluded lesions without pathological confirmation which led to a higher PPV in category 4a.

The relationship between breast density reflected by breast composition and PPV in subcategories 4a, 4b, and 4c were not statistically significant and showed no positive correlation between increasing density and PPV. The National Breast and Ovarian Cancer Center of Australia (2019) reported women who had the highest degree of breast density from mammograms were at 4–6 times greater risk for breast cancer than no breast density, but recent reviews found that this correlation was controversial (Momenimovahed and Salehiniya, 2019). This negative finding may be from the qualitative assessment fashion of the BI-RADS lexicon which focuses on the potential masking effect of dense breast tissue on a lesion instead of the volume of fibroglandular tissue which increases the risk of breast cancer (Destounis et al., 2017). Another reason that caused difficult interpretation was a fewer populations in the entirely fat (1.87%) and extremely dense (2.71%) types were compared to the other two type of breast composition.

This study had some limitations. First, the ACR BI-RADS lexicon 5th edition was not widely used during the years of 2016 and 2017 among our radiologists which caused category 4 (19.97%) to be reported. Second, since the data were collected retrospectively from the official reports in the Hospital Information System, the inter-observer reliability was not assessed. Third, since benign lesions without biopsy-proven were included, selection bias might be a concern. Finally, the radiologists were not blinded to the age of the patients during the examination which meant some bias possibly occurred.

There was a significantly positive association between PPV and age in patients with lesions of BI-RADS subcategories 4a, 4b, and 4c. Age-related PPV in young patients (<35 years) was lower than the standard reference. This study could not determine that mammographic breast composition according to the ACR BI-RADS 5^th ^edition was associated with a PPV due to improper sample distribution.

## References

[B1] American College of Radiology (2013). Breast Imaging Reporting and Data System (BI-RADS).

[B2] Bae JM, Kim EH (2016). Breast density and risk of breast cancer in Asian women: A Meta-analysis of Observational Studies. J Prev Med Public Health.

[B3] Bashar MD A, Aggarwal AK (2020). A successful model of cancer screening in low resource settings: Findings of an Integrated Cancer Screening Camp from a Rural Setting of North India. Asian Pac J Cancer Care.

[B4] Bond M, Pavey T, Welch K (2013). Systematic review of the psychological consequences of false-positive screening mammograms. Health Technol Assess.

[B5] Burivong W, Amornvithayacharn O (2011). Accuracy of subcategories A, B, C in BI-RADS 4 lesions by combined mammography and breast ultrasound findings. Med Med Sci.

[B6] Chaiwerawattana A, Thanasitthichai S, Boonlikit S (2012). Clinical outcome of breast cancer BI-RADS 4 lesions during 2003-2008 in the National Cancer Institute Thailand. Asian Pac J Cancer Prev.

[B7] Destounis S, Arieno A, Morgan R (2017). Qualitative versus quantitative mammographic breast density assessment: Applications for the US and Abroad. Diagnostics.

[B8] D’Orsi C, Sickles EA, Mendelson EB (2013). Breast Imaging Reporting and Data System: ACR BI-RADS breast imaging atlas.

[B9] Elezaby M, Li G, Bhargavan-Chatfield M (2018). ACR BI-RADS assessment category 4 subdivisions in diagnostic mammography: Utilization and Outcomes in the National Mammography Database. Radiology.

[B10] Flowers CI, O’Donoghue C, Moore D (2013). Reducing false-positive biopsies: a pilot study to reduce benign biopsy rates for BI-RADS 4A/B assessments through testing risk stratification and new thresholds for intervention. Breast Cancer Res Treat.

[B11] Fu C-Y, Hsu2 H-H, Yu J-C (2011). Influence of age on PPV of sonographic BI-RADS categories 3, 4, and 5. Ultraschall Med.

[B12] He P, Cui LG, Chen W (2019). Subcategorization of Ultrasonographic BI-RADS Category 4: Assessment of Diagnostic Accuracy in Diagnosing Breast Lesions and Influence of Clinical Factors on Positive Predictive Value. Ultrasound Med Biol.

[B13] Hu Y, Yang Y, Gu R (2018). Does patient age affect the PPV3 of ACR BI-RADS Ultrasound categories 4 and 5 in the diagnostic setting?. Eur Radiol.

[B14] Imsamran W, Pattatang A, Supattagorn P (2018). Songkhla Cancer Registry. Cancer Thailand.

[B15] Kotepui M, Chupeerach C (2013). Age distribution of breast cancer from a Thailand population-based cancer registry. Asian Pac J Cancer Prev.

[B16] Lazarus E, Mainiero MB, Schepps B (2011). BI-RADS lexicon for US and mammography: Interobserver Variability and Positive Predictive Value. Radiology.

[B17] Mazouni C, Sneige N, Rouzier R (2010). A nomogram to predict for malignant diagnosis of BI-RADS Category 4 breast lesions. J Surg Oncol.

[B18] McCarthy AM, Keller B, Kontos D (2015). The use of the Gail model, body mass index and SNPs to predict breast cancer among women with abnormal (BI-RADS 4) mammograms. Breast Cancer Res.

[B19] Momenimovahed Z, Salehiniya H (2019). Epidemiological characteristics of and risk factors for breast cancer in the world. Breast Cancer (Dove Med Press).

[B22] Nelson HD, Fu R, Cantor A (2016). Effectiveness of Breast Cancer Screening: Systematic Review and Meta-analysis to Update the 2009 U Preventive Services Task Force Recommendation. Ann Intern Med.

[B23] Nindrea RD, Aryandono T, Lazuardi L (2017). Breast cancer risk from modifiable and non-modifiable risk factors among women in Southeast Asia: A Meta-Analysis. Asian Pac J Cancer Prev.

[B24] Pettersson A, Graff RE, Ursin G (2014). Mammographic density phenotypes and risk of breast cancer: a meta-analysis. J Natl Cancer Inst.

[B25] Rojanamethin J, Seangkrajang S, Seangariyavanich A (2020). Hospital-Based Cancer Registry.

[B26] Spak DA, Plaxco JS, Santiago L (2017). BI-RADS((R)) fifth edition: A summary of changes. Diagn Interv Imaging.

[B27] Suttawas A (2018). Positive Predictive Value and Biopsy Rate of Breast Cancer in BI-RADS Category 4 and 5 Breast Lesions. Reg 4-5 medl j.

[B28] Weir R, Day P, Ali W (2007). Risk factors for breast cancer in women: a systematic review of the literature. NZHTA Report.

[B29] Wiratkapun C, Bunyapaiboonsri W, Wibulpolprasert B (2010). Biopsy Rate and Positive Predictive Value for Breast Cancer in BI-RADS Category 4 Breast Lesions. J Med Assoc Thai.

[B30] Yoon JH, Kim MJ, Moon HJ (2011). Subcategorization of ultrasonographic BI-RADS category 4: positive predictive value and clinical factors affecting it. Ultrasound Med Biol.

